# The socioeconomic gradient in mortality from ovarian, cervical, and endometrial cancer in Australian women, 2001–2018: A population‐based study

**DOI:** 10.1111/ajo.13553

**Published:** 2022-06-16

**Authors:** James Gregory, Leon Foster, Pauline O'Shaughnessy, Stephen J. Robson

**Affiliations:** ^1^ Junior Medical Officer Nepean Hospital Sydney New South Wales Australia; ^2^ Senior Subspecialty Trainee, Gynaecological Oncology Royal Hospital for Women Sydney New South Wales Australia; ^3^ School of Mathematics and Applied Statistics University of Wollongong Wollongong New South Wales Australia; ^4^ School of Health and Medicine Australian National University Canberra Australian Capital Territory Australia

**Keywords:** cancer, ovary, cervix, endometrium, socio‐economic

## Abstract

**Background:**

Socio‐economic (SE) status is closely linked to health status and the mechanisms of this association are complex. One important adverse effect of SE disadvantage is vulnerability to cancer and cancer is a major cause of morbidity and mortality in Australia.

**Aims:**

We aimed to estimate the effect of SE status on mortality rates from ovarian, cervical, and endometrial cancer.

**Materials and Methods:**

National mortality data were obtained from the Australian Bureau of Statistics (ABS) for the calendar years from 2001 to 2018, inclusive. Individual deaths were grouped by the ABS Index of Relative Socio‐economic Advantage and Disadvantage. Population data were obtained to provided denominators allowing calculation of mortality rates (deaths per 100 000 women aged 30–79 years). Statistical analyses performed included tabulating point‐estimates of mortality rates and their changes over time and modelling the trends of rates using maximum likelihood method.

**Results:**

Age‐standardised mortality rates for ovarian and cervical cancer fell over the study period but increased for endometrial cancer. There was clear evidence of a SE gradient in the mortality rate for all three cancers. This SE gradient increased over the study period for ovarian and cervical cancer but remained unchanged for endometrial cancer.

**Conclusions:**

Women at greater SE disadvantage have higher rates of death from the commonest gynaecological cancers and this gradient has not reduced over the last two decades. After the COVID‐19 pandemic efforts must be redoubled to ensure that Australians already at risk of ill health do not face even greater risks because of their circumstances.

## INTRODUCTION

It has long been recognised that an individual's socio‐economic (SE) status – their family circumstances, housing, working conditions, income, and education – is closely linked to their health status.^1^ The mechanisms through which SE status affects health are complex and involve differences in exposures and vulnerabilities: adverse outcomes commonly manifest over a long period of time. Unfortunately, disparities in SE status have only increased in Australia during the COVID‐19 pandemic, meaning that SE‐related adverse health outcomes are likely to lead to further pressures on health and health systems.^2^ One important adverse effect of SE disadvantage is vulnerability to cancer. Australian data reveal that the incidence rate of cancers is highest – and survival rates are lowest – in areas of the greatest social and economic disadvantage.^3^ This association between SE status and risk of cancer has been globally observed.^4^, ^5^

Cancer is a major cause of morbidity and mortality in Australia: malignant conditions account for approximately three of every ten deaths.^3^ Unsurprisingly cancer has a major effect on the Australian economy with the direct costs of providing cancer‐related medical care to Australians comprising about 0.5% of the gross domestic product (GDP).^6^ In addition there are economic effects associated with lost productivity due to early loss of life estimated to amount to greater than $4 billion yearly in Australia,^7^ and lost productivity from cancer treatment has been estimated at close to $2 billion each year.^8^

An individual's risk of an adverse outcome of cancer is generally associated with multiple factors: their risk of developing a malignant disease; the stage at diagnosis; co‐morbidities in affected individuals; and, access to good‐quality medical care in managing disease.^9^ However, Australia provides an example where a government‐funded universal health system is available which, in theory at least, provides equity in access at a population level: internationally, Australia's healthcare system and its outcomes rank within the top ten countries for healthcare efficiency.^10^ Taking into account this system of ‘universal’ healthcare we aimed to estimate the effect of SE status on mortality rates from the three commonest gynaecological cancers – ovarian, cervical, and endometrial – in the pre‐COVID‐19 era.

## METHODS

National mortality data for endometrial, ovarian, and cervical cancer were obtained from the Australian Bureau of Statistics (ABS) for the calendar years from 2001 to 2018 inclusive. The ABS carries national responsibility for compiling the national data on deaths in Australia. Mortality data are obtained from all Australian state and territory Registrars of Births, Deaths and Marriages, and the notifications are supplemented by information from the National Coroners Information System (NCIS). Information about an individual's cause of death as used by the ABS is supplied by the medical practitioner who certifies the death or, in certain circumstances, by a coroner. The ABS uses the *International Classification of Diseases – Version 10 (ICD‐10)* for coding of cause of death.^11^ The ABS website publishes detailed information about the data evaluation process.^12^

For our measure of SE status we used the ABS classification of Socio‐Economic Indexes for Areas (SEIFA) which ranks areas in Australia according to relative SE advantage and disadvantage and is based on information from the five‐yearly Australian National Census.^13^ The SEIFA classification system has two ‘general’ indices and two additional indices, one each for occupation and education. Of the available SEIFA indices the most relevant for our analysis is the SEIFA Index of Relative Socio‐economic Advantage and Disadvantage (IRSAD). The IRSAD includes measures of family and social status such as the proportion of one‐parent families, access to transport, occupational and educational status, and available economic resources. The specific measures in each index are listed in the reports from the ABS.^13^

Population data were obtained from the ABS to allow the calculation of the total number of women aged 30–79 years (the age group in which 95% of mortality occurs), later used as the denominator in calculating mortality rates. We used point‐estimates for June of each year of the study according to Statistical Areas Level 2 (SA2) which are medium‐sized ‘general purpose’ to represent a community that interacts together socially and economically.^14^ There are 2310 SA2 regions covering the whole of Australia without gaps or overlaps. For each year of the study, an estimate was obtained of the number of women in each SEIFA quintile to allow calculation of mortality rates (as deaths per 100 000 women aged 30–79 years in each SEIFA decile) specific to cause of death. Statistical analysis performed includes tabulating the changes of mortalities over years across different SEIFA groups and modelling the trends of rates using maximum likelihood estimation method. The study received prospective approval from the Australian National University Human Research Ethics Committee (Protocol 2021/092).

## RESULTS

**Figure 1:**
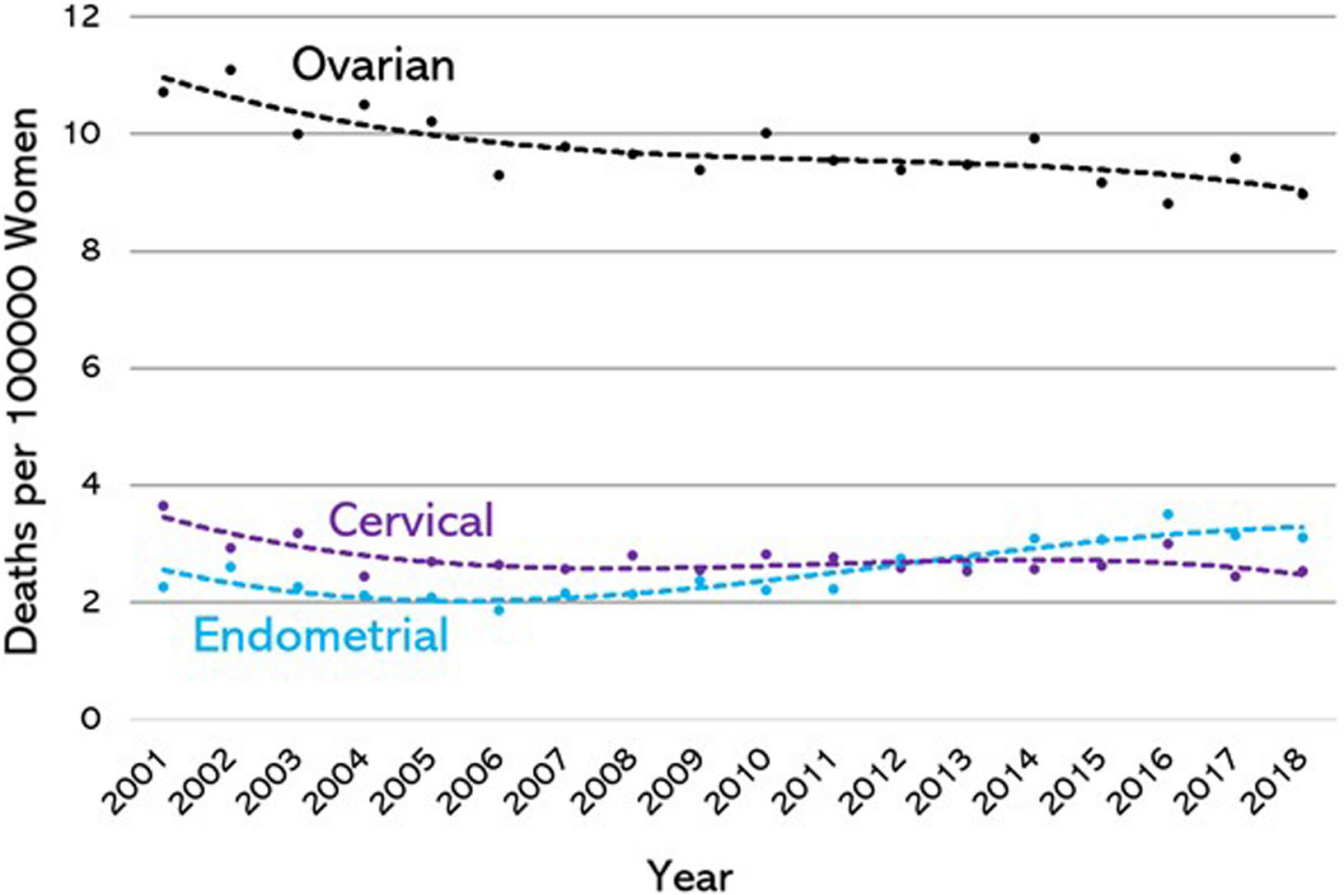
Overall mortality rates (deaths per 100 000 per year) for ovarian, cervical, and endometrial cancer in Australian women aged 30–79 years, age stratified, from 2001 to 2018 (inclusive).

The overall mean age‐standardised rate of mortality (ASR, expressed as the number of deaths per 100 000 women) for ovarian cancer over the study period (9.71) was considerably higher than that of cervical (2.72) and endometrial (2.56) cancer over the study period (Fig. 1). However, while the ASR fell both for ovarian and cervical cancer between the first triennium of the study (2001–2003) and the last triennium (2016–2018), the ASR for endometrial cancer increased. The ASR for ovarian cancer fell from 10.6 to 9.1, a reduction of 14.2%, and for cervical cancer it fell from 3.24 to 2.64, a reduction of 18.5%. The ASR for endometrial cancer increased from 2.37 to 3.25, an increase of 37.1%.

**Figure 2:**
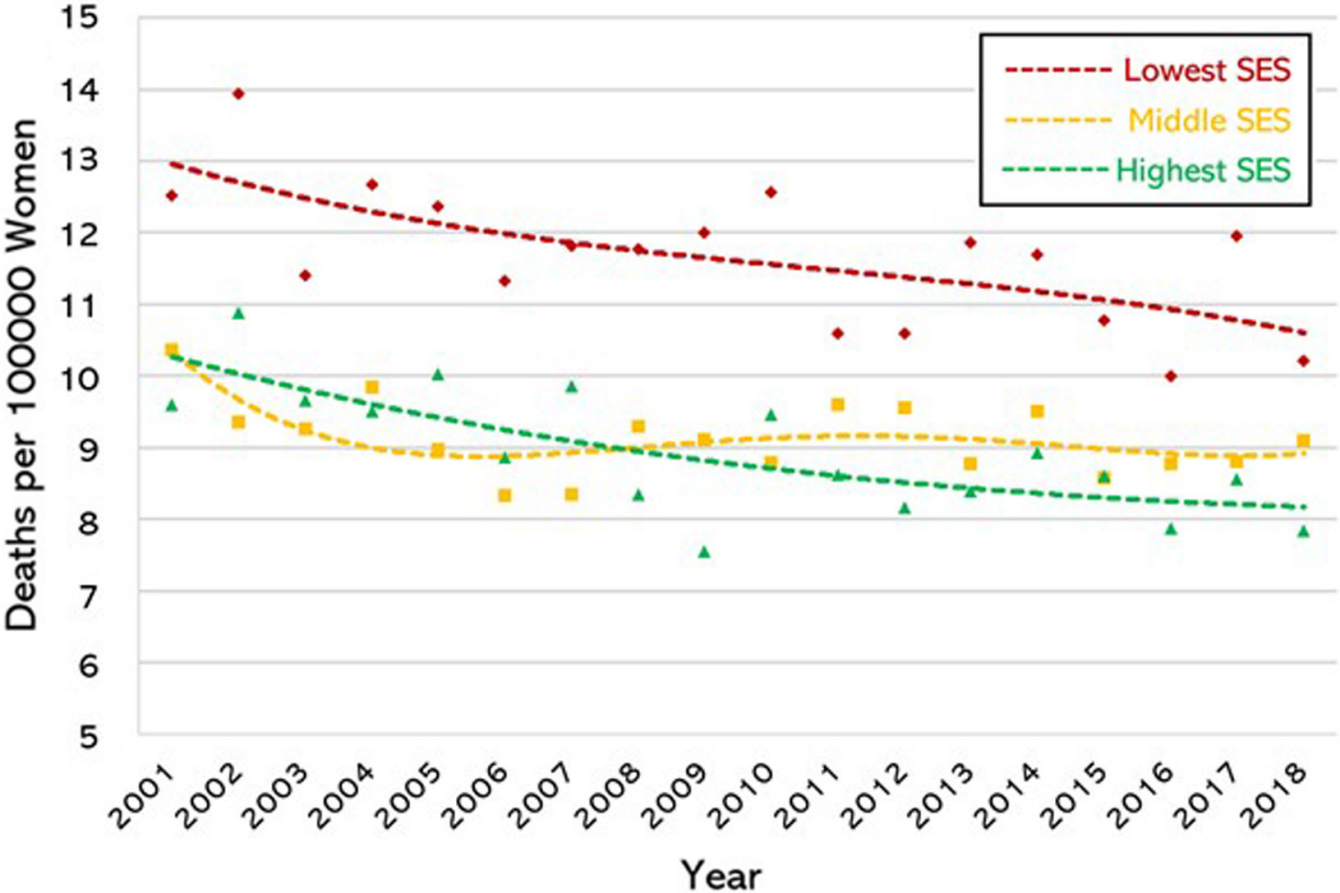
Ovarian cancer mortality rate (deaths per 100 000 per year) in Australian women aged 30–79 years, age stratified, from 2001 to 2018 (inclusive) according to socio‐economic status (SES) using Australian Bureau of Statistics Index of Relative Socio‐economic Advantage and Disadvantage deciles. Lowest three deciles (♦), middle four deciles (■), and highest three deciles (▲).

**Figure 3:**
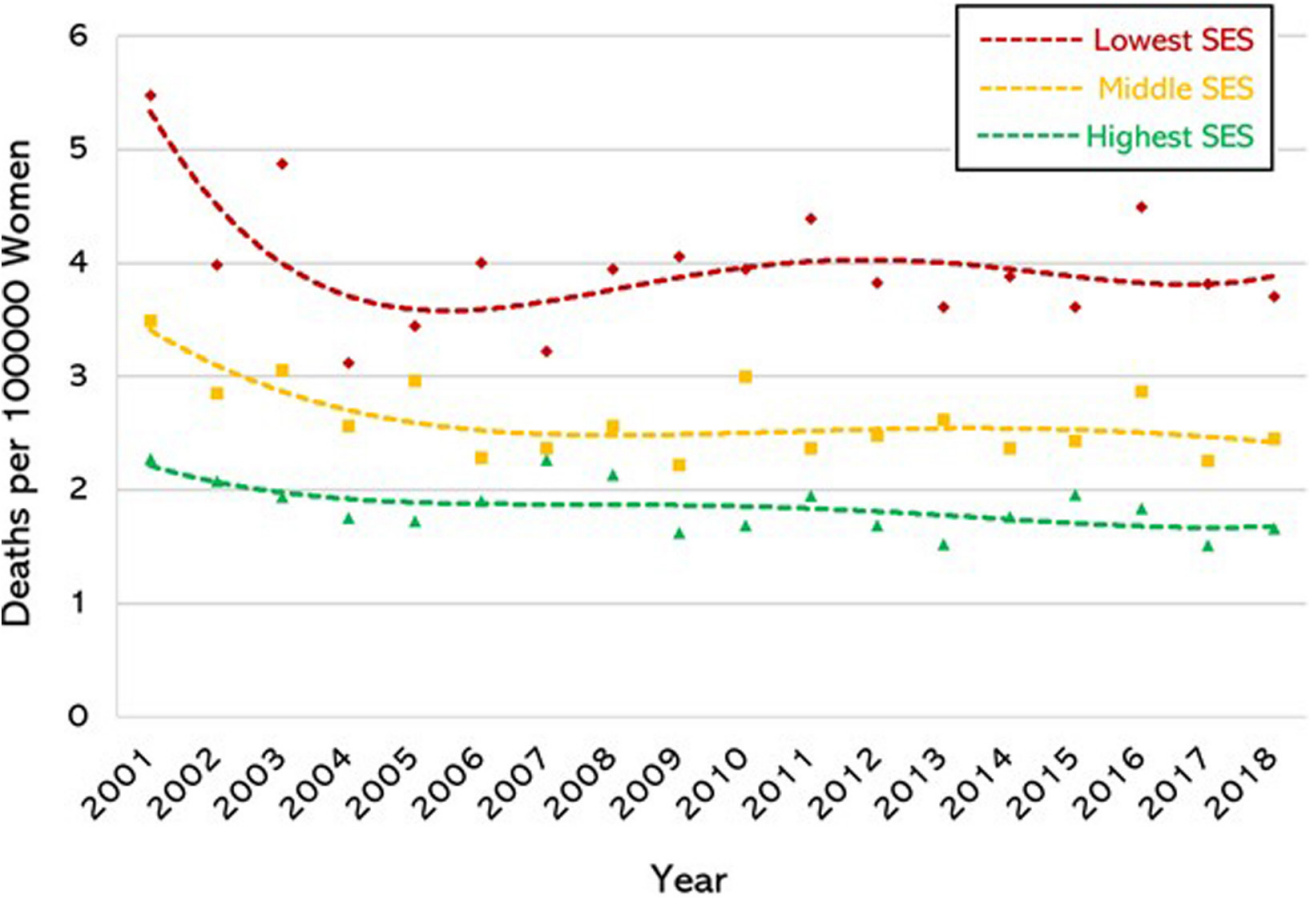
Cervical cancer mortality rate (deaths per 100 000 per year) in Australian women aged 30–79 years, age stratified, from 2001 to 2018 (inclusive) according to socio‐economic status (SES) using Australian Bureau of Statistics Index of Relative Socio‐economic Advantage and Disadvantage deciles. Lowest three deciles (♦), middle four deciles (■), and highest three deciles (▲).

**Figure 4:**
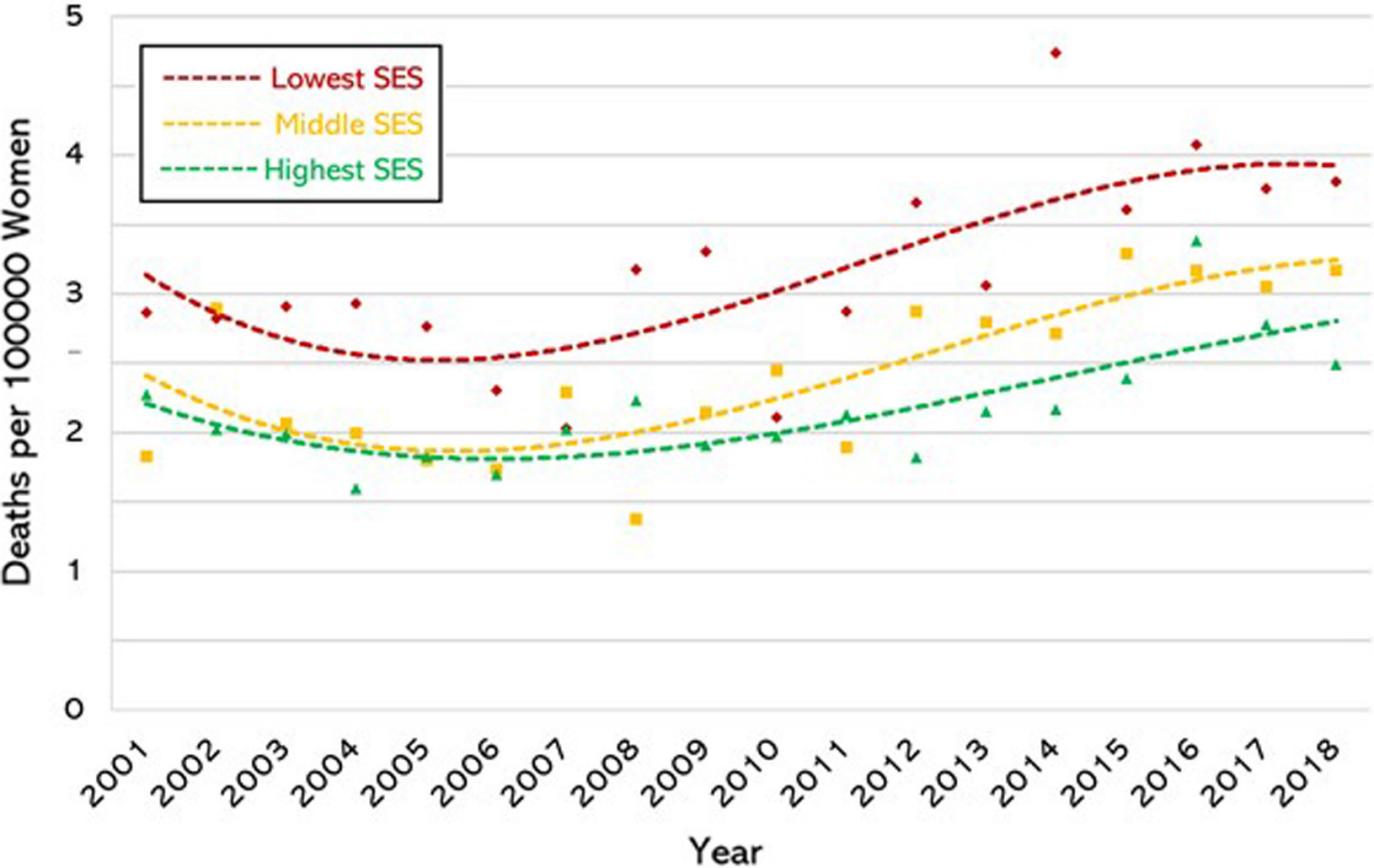
Endometrial cancer mortality rate (deaths per 100 000 per year) in Australian women aged 30–79 years, age stratified, from 2001 to 2018 (inclusive) according to socio‐economic status (SES) using Australian Bureau of Statistics Index of Relative Socio‐economic Advantage and Disadvantage deciles. Lowest three deciles (♦), middle four deciles (■), and highest three deciles (▲).

There was clear evidence of a SE gradient in the mortality rate for each individual cancer. To identify differences in ASR according to ecological SE status of the cohort residence we analysed the data in three groups: women at greatest SE disadvantage (IRSAD deciles 1–3, inclusive), women at moderate SE disadvantage (IRSAD deciles 4–7, inclusive) and women in the highest SE groups (IRSAD deciles 8–10, inclusive). There was a clear and significant SE gradient for all three malignancies: ovarian (Fig. 2); cervical (Fig. 3); and, endometrial (Fig. 4). Of note, the SE gradient in mortality rates increased over the study period for ovarian and cervical cancer but remained unchanged for endometrial cancer (Table 1).

**Table 1:** Comparison of ASR of mortality (deaths per 100 000 women aged 30–79 years, inclusive) for each malignancy between the first and last triennia of the study.

2001–2003				2016–2018			
	Lowest SE	Highest SE	ASR gradient (%)	Lowest SE	Highest SE	ASR gradient (%)	*P*‐value
Ovarian	12.61	10.05	+2.57 (25.5%)	10.72	8.09	+2.63 (32.5%)	<0.005
Cervical	4.77	2.10	+2.68 (127.6%)	4.00	1.67	+2.33 (139.4%)	<0.005
Endometrial	2.87	2.10	+0.77 (36.7%)	3.88	2.88	+1.00 (34.8%)	n.s.

Note: The gradient is the additional number of deaths per 10 000 women in the groups.

ASR, age‐standardised rate; SE, socio‐economic status.

Mortality from ovarian cancer decreased across all SE groups over the study period (Fig. 2). We identified a marked socio‐economic gradient with women in the lowest socio‐economic groups having a higher mortality rate across the study period. Mortality from endometrial cancer showed a similar socio‐economic gradient (Fig. 3). Importantly, endometrial cancer is the only example in our study where the mortality increased over the study period. When we examined the rate of increase in mortality we found that all SE status quintiles showed similar quadratic growth in mortality, and there was no difference in the rate of change in the different SE groups (*P* = 0.198 and *P* = 0.386 for differentiating linear and quadratic components among quintiles respectively).

Over the study period, cervical cancer mortality showed a net decrease across all socio‐economic groups (Fig. 4). The rate of fall was uneven (*P* = 0.006) due to the lowest SE groups experiencing the largest decrease in mortality rates – from 0.6 to 0.4/100 000 – with large year‐to‐year fluctuations prior to 2009. After 2009 the mortality rate for the most disadvantaged women remained stable. The other SE groups showed gradual decrease status over the study period.

## DISCUSSION

In this study we have demonstrated a significant gradient in mortality rates for the three commonest gynaecological cancers and, importantly, that this disparity in mortality between women at greatest and least SE disadvantage was increasing prior to the COVID‐19 pandemic. A limitation of this study is that it was not possible to adjust for co‐morbidities and staging at diagnosis. However, the focus of the study was not on modelling individual effects but to provide a population‐level estimate of the ecological effect of SE status on cancer mortality rates and to determine whether these effects were increasing or narrowing over time.

Inequality and entrenched disadvantage have a negative effect on social cohesion and population wellbeing. The Australian Productivity Commission (APC) has studied inequality, poverty and disadvantage in Australia using a number of metrics including the Gini coefficient.^15^ The Gini coefficient is an economic measure of income and wealth inequality within a country that varies between 0 and 1, where the lower the value the more equitable the wealth distribution within a country's population. The APC concluded that inequality in Australia was slowly increasing with Australia's current Gini coefficient of 0.33 placing us below the Organisation for Economic Co‐operation and Development average – ahead of the United Kingdom and United States, for example, but less equitable than Canada, France, and Scandinavian countries. Increased SE inequality is an important metric, since a person's SE status influences cancer outcomes in multiple ways. Individuals at greater SE disadvantage are more likely to be affected by risk factors for cancer such as smoking, obesity, and poorer nutrition.^16^ Lower SE status also is associated with reduced access to cancer prevention and screening programs.^17^ Once a cancer diagnosis is established SE status influences an individual's access to cancer therapies such as high‐quality surgery, adjuvant chemo‐ and radiotherapy treatments, as well as longer‐term survivor surveillance for recurrent disease.^18^

The highest mortality rate was for ovarian cancer, the fifth most common cancer in Australian women.^3^ It is already well recognised that SE status influences survival in ovarian cancer^19^ and this has been at least partially attributed to a tendency to later‐stage disease at the time of diagnosis in women at greater SE disadvantage.^20^ A study from the United States reported that greater SE disadvantage was associated with a higher probability of suboptimal debulking, higher stage at diagnosis, and higher histological tumour grade.^21^ Data from a Chinese study of ovarian cancer covering the period 1983–2012 reported population‐level findings similar to our results with a reduction in incidence rate and a widening of mortality gradient across SE groups.^22^ At a global level there is evidence from systematic reviews that women at SE disadvantage commonly lacked access to ‘quality ovarian cancer treatment’ and, as a result, mortality rates were higher.^23^

In many respects cervical cancer is a prototype disease for which SE inequality is a major risk factor. Cervical cancer is a less common disease for Australian women now, ranking as the 14th most commonly diagnosed cancer by gender with fewer than 1000 new diagnoses of invasive disease each year.^3^ However, globally cervical cancer is the fourth most frequently occurring malignancy leading to 270 000 deaths annually. This disease primarily affects younger women with the greatest burden of disease in low‐ and middle‐income countries where the mortality rate is 18 times greater than in high‐income countries.^24^ Cervical cancer is a largely preventable disease: Australia hosts an effective national program of human papilloma virus (HPV) vaccination and cervical screening. We found the mortality rate of cervical cancer to be 1.3 times higher for woman at the greatest SE disadvantage and this was the malignancy with the largest mortality gradient.

Commencement of the HPV vaccination in Australia in 2007 has led to vaccine coverage rates in women of up to 85%.^25^, ^26^ SE factors have influenced participation in population screening programs and this effect has been recognised for many years.^27^ The majority of cervical cancers diagnosed in Australian women occur in those who are under‐screened or have never been screened. With the ‘refresh’ of Australian cervical screening program and transition to HPV‐based screening with reflex cytology, self‐collected specimens have been introduced and there is anticipation that this technology could increase participation in screening.^28^

The risk factors for endometrial cancer have been difficult to disentangle and a recent comprehensive review of the literature concluded that increased body mass index (BMI), nulliparity, and diabetes were most strongly associated with increased risk.^29^ Another systematic review that specifically dealt with survival reported that SE deprivation was associated with a reduction in survival; however, it remained unclear whether this effect was mediated by BMI, smoking, and other co‐morbidities.^30^ Obesity is closely associated with SE disadvantage^31^ and it is possible that this association explains the link between SE status and development of endometrial cancer, although it is less clear how this would influence survival. The most likely reason for increasing mortality from endometrial cancer is the increased rate of overweight and obesity in Australia, as is occurring overseas in developed countries.^32^ As many as half of all endometrial cancers are attributable, in full or in part, to obesity^33^ and obesity has been associated with a roughly twofold increase in endometrial cancer‐specific mortality.^34^ There is a well‐recognised gradient in the incidence of obesity according to SE status with women in lower SE groups, and more recent birth cohorts, at higher risk of obesity and severe obesity.^35^

This population‐based study has shown that mortality rates for the three most common gynaecological cancers follow a gradient with women at greater SE disadvantage having higher rates of death. Importantly, the gradient has not reduced over the last two decades and, indeed, seems to have increased. An individual's risk of cancer is strongly influenced by their SE status, but so too are many other aspects of health. Even before the COVID‐19 pandemic there is evidence that social and health inequality are increasing in Australia. In the aftermath of the pandemic it is important that efforts are redoubled to ensure that Australians already at risk of ill health do not face even greater risks because of their circumstances.
